# Liquid Metal Embrittlement Susceptibility and Crack Formation of the Zn-Coated Complex Phase Steel

**DOI:** 10.3390/ma18010009

**Published:** 2024-12-24

**Authors:** Rongxun Hu, Jiayi Zhou, Yu Sun, Ming Lei, Yulai Gao

**Affiliations:** 1 State Key Laboratory of Advanced Special Steel, School of Materials Science and Engineering, Shanghai University, Shanghai 200444, China; rongxunhu@shu.edu.cn (R.H.); zhoujy00@shu.edu.cn (J.Z.); sunyu00@shu.edu.cn (Y.S.); 2 State Key Laboratory of Development and Application Technology of Automotive Steels, Shanghai 201900, China; 3 Automobile Steel Research Institute, R&D Center, Baoshan Iron & Steel Co., Ltd., Shanghai 201900, China

**Keywords:** liquid metal embrittlement (LME) susceptibility, complex phase (CP) steel, advanced high-strength steels, resistance spot-welding, internal oxide layer

## Abstract

In the resistance spot-welding (RSW) of galvanized complex phase (CP) steel, liquid metal embrittlement (LME) may occur, deteriorating the welded joint’s performance. Based on the Auto/Steel Partnership (A/SP) standard, the joints of galvanized CP steel welded with a welding current from 7.0 kA to 14.5 kA were evaluated. When the welding current increased to 11.0 kA, LME cracks began to appear. The longest type A crack was 336.1 μm, yet the longest type D crack was 108.5 μm, and did not exceed 10% of the plate thickness, which met the limitation of the A/SP standard. In light of the microstructural observation and element distribution, it was found that there existed an internal oxide layer adjacent to the surface of galvanized CP steel matrix, with the depth of about 4.1 μm. In addition, the simulation results show that the CP steel was under tensile stress throughout the RSW process, but the internal oxide layer could successfully lead to the low LME susceptibility of the Zn-coated CP steel.

## 1. Introduction

In recent years, the challenges of energy shortage and environmental contamination have become increasingly prominent [1,2]. Correspondingly, the automotive industry has actively responded to the requirements, and lightweight automobiles have attracted much more attention, aiming to achieve the purpose of energy saving and emission reduction [3]. To reduce the weight of the car while ensuring the strength and safety, advanced high-strength steels (AHSSs) have already been extensively utilized [4]. The high strength can effectively produce good energy absorption, which plays an important role in lightweight automobiles [5,6]. Thus, excellent performance can be realized in car body, reinforcement parts and safety, such as A/B/C pillars, door anti-collision beams and seat rails, etc. [7,8]. Advanced high-strength steels mainly include complex phase (CP) steel, dual-phase (DP) steel, transformation-induced plasticity (TRIP) steel [9,10], twinning-induced plasticity (TWIP) steel [11], and quenching-partitioning (QP) steel, etc. [12]. Among these, CP steels can offer an excellent combination of high strength, ductility and formability, making them an attractive alternative to those conventional steels. In particular, the microstructure of CP steel is composed of ferrite, bainite, and residual austenite. Compared to the DP steel, CP steel possesses higher tensile strength and yield strength, exhibiting excellent energy absorption and high residual strain capacity [13,14,15]. Generally, in order to improve the corrosion resistance of AHSS and extend its service life, a galvanized layer needs to be coated on the surface of AHSS [16]. Generally, three methods are extensively employed to obtain the Zn coating of the AHSSs: hot-dip galvanizing (GI), galvannealing (GA), and electro-galvanizing (EG). Among them, the galvanizing technique is widely used due to its high efficiency and lower cost [17]. Although the Zn coating could delay the corrosion of AHSS, it could also bring about the issue of liquid metal embrittlement (LME) during the resistance spot-welding (RSW) process, dramatically limiting the application of galvanized AHSS in the manufacturing process [18].

Spot-welding is an important joining process in production, such as gas tungsten arc spot-welding, laser spot-welding, and friction stir spot-welding, etc. [19]. Among them, resistance spot-welding is widely used by virtue of its advantages of high efficiency, stability, automation and low cost [20]. As a typical welding technique, resistance spot-welding could generate a large amount of heat when the current is transported through resistance [21]. A nugget is then formed, and the steel plates could be joined with each other under the applied force of the electrodes [22]. As a common reliable and efficient connection technique, RSW has been extensively used in the connection of automobile parts. Unfortunately, cracks are possibly produced in the process of resistance spot-welding for galvanized steel plates due to the penetration of liquid Zn along the grain boundaries, which is defined as liquid metal embrittlement [23,24,25].

LME is an abnormal phenomenon, and usually appears in some specific solid–liquid metal systems under external stress [26,27]. During LME crack formation, the liquid metal can enter the solid metal along the grain boundaries, and finally lead to intergranular cracking [28,29,30]. In the process of RSW, the initiation and propagation of LME cracks could be triggered by various factors, including electrode stress, type of steels, and composition of coating, etc. [31,32,33]. Pant et al. [34] found that the levels of LME susceptibility in three kinds of martensitic steels with the same tensile strength were different because of their various microstructures and prior austenitic grain sizes. Beal et al. [16] studied TWIP steel with a high manganese content, and noticed that the LME cracks only occurred at a certain temperature. Moreover, El-Sari et al. [35] comparatively investigated the severity of LME for steels with different coatings, and found that the degrees of LME susceptibility of electrogalvanized, galvannealed and galvanized coatings were significantly less than that of the zinc–magnesium coatings. Kim et al. [9] proved that for TRIP steel plates, electrogalvanized coatings could produce the strongest resistance to LME, yet the continuously galvanized Zn coating revealed the highest LME susceptibility.

Considering the liquid metal embrittlement caused by Zn in the process of resistance spot-welding, some researchers have developed a variety of methods to reduce its formation [36,37]. Kim et al. [38] found that pre-current treatment before the second incoming current could obviously reduce weld surface cracks even under expulsion current conditions. He et al. [39] deemed that element Al could suppress the LME by forming iron aluminides, with the Al intermediate layer being added between the electrode and the steel plate.

Complex phase steel is one of the typical advanced high strength steels [40,41]. It has been widely used in the production of automotive parts such as chassis suspension components, B-pillar reinforcement plates and seat slides, due to its excellent properties [42]. Among the multiphase steels, CP steel has attracted increasing attention from the industry, attributed to its improved local formability as compared to others [43,44]. As the protection layer, Zn coating is required to increase the corrosion resistance of the steel substrate. Nevertheless, little information is available regarding the LME susceptibility of the Zn-coated CP steel, which needs to be evaluated systematically. Based on the above-mentioned considerations, the LME susceptibility of the galvanized CP steel was evaluated based on the Auto/Steel Partnership (A/SP) standard [45]. The details of the LME cracks were observed and analyzed, and the most influential factors were discussed in the present study. In addition, the microstructure and element distribution of the welded joints were observed. Based on the results regarding the microstructure of the jointed Zn-coated steel plates combined with the coating details, the LME susceptibility in the galvanized CP steel was systematically analyzed and discussed.

## 2. Materials and Experiments

The chemical composition of the CP steel was analyzed with the inductively coupled plasma atomic emission spectrometry (ICP-AES, Agilent 5110, Santa Clara, CA, USA), as listed in Table 1. After hot dip galvanizing, a zinc coating with a thickness of about 20 μm was obtained. The Zn-coated CP steel with a thickness of 1.4 mm was welded by the applied RSW technique. During the welding process, the experiment was carried out based on the A/SP standard, which was extensively employed as the evaluation criterion. According to the A/SP standard [45], 8 stages of gradually increasing inclined pulse currents were employed to join two steel plates. In this study, 16 groups of the welded joints at different welding currents were designed. The commencing welding current was 5.0 kA and the end welding current was set from 7.0 kA to 14.5 kA, with an interval of 0.5 kA. The duration of each pulse was 130 ms and the interval between the two pulses was 40 ms. The electric force was 4.0 kN, with a squeeze time 1000 ms and holding time 250 ms. The specific crack classification was presented with a clearer schematic image in our previous study [45]. A crack in the center of the electrode indentation was defined as type A. A type D crack was located at the shoulder of the indentation. Type B and C cracks could be observed outside the electrode indentation and between the two steel plates, respectively. The whole experiment was carried out based on the A/SP standard, thus such an evaluation could objectively reflect the LME susceptibility of the welded joint, with better repeatability.

**Figure 1:**
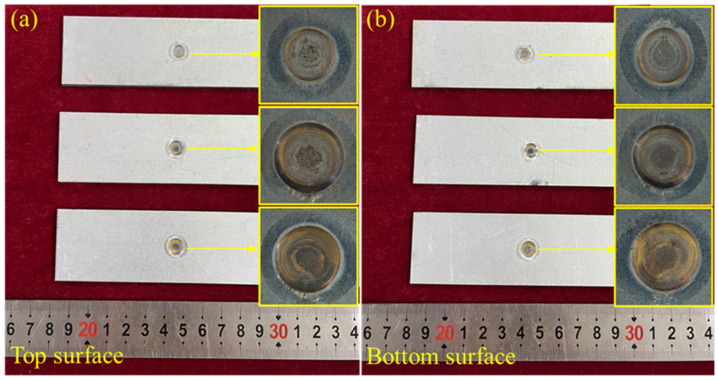
Appearance of the three typical welded joints in the conditions below expulsion, at expulsion and above expulsion: (**a**) top surface of the two jointed steel plates, and (**b**) bottom surface of the two jointed steel plates. The insets show magnified images of the RSW welded zones, revealing their detailed appearance.

In general, the case of expulsion at the minimum welding current was defined as the condition at expulsion [46]. During the RSW process, expulsion occurred with a welding current of 11.0 kA. Thus, three typical samples welded with the currents of 10.5 kA, 11.0 kA and 13.0 kA were chosen for the further microstructure analysis, as shown in Figure 1, corresponding to the sample conditions below expulsion, at expulsion and above expulsion, respectively. The insets in Figure 1 show magnified images of the welded joints, revealing the appearance in detail.

A static tensile testing machine (Instron 5581, Norwood, MA, USA) was used to measure the mechanical properties of the CP steel at a tensile rate of 0.001 s^−1^. In order to study the LME crack tendency during the resistance spot-welding of the samples under different currents, the longest crack was traced and measured on the removed Zn surface of the RSW joints. Therefore, to much more precisely identify the morphology of the longest LME crack, (CH_2_)_6_N_4_-HCl (5 g (CH_2_)_6_N_4_ was added into 250 mL hydrochloric acid, and then deionized water was added until the volume reached 500 mL. This solution was used to remove the Zn coating on the surface of the welded joints. By controlling the etching time appropriately, the longest crack could be clearly revealed. Subsequently, the samples were ground and polished in the direction perpendicular to the maximum crack observed from the removed Zn surface. By these means, the possibility of missing the longest crack could be reduced to some extent. The polished samples were observed with an optical microscope (OM, Zeiss Axioscope 5, Jena, Germany). The microstructure of the welded joints and thickness of the coating were observed by scanning electron microscope (SEM, Zeiss GeminiSEM 300, Jena, Germany). The microhardness of the sample was determined by a Vickers microhardness tester (AHVST-1000ZXY, Shanghai, China). The element distribution was detected using an electron probe micro-analyzer (EPMA, JXA-iHP200F, Tokyo, Japan). The SORPAS 2D Welding (15.30, SWANTEC, Copenhagen, Denmark) software was used to simulate the temperature and stress distribution of the RSW process.

## 3. Results and Discussion

**Figure 2:**
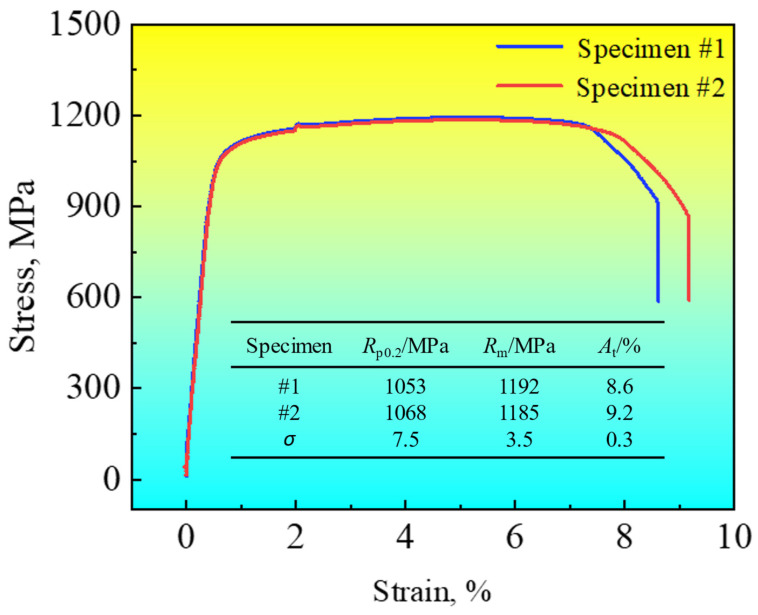
The engineering stress–strain curves of the as-prepared galvanized CP steels.

The engineering stress–strain curves of two galvanized CP steels are illustrated in Figure 2. In specimen #1, the yield strength (YS), ultimate tensile strength (UTS) and percentage of elongation (EL) were 1053 MPa, 1192 MPa and 8.6%, respectively. For the other specimen, the YS, UTS and EL were 1068 MPa, 1185 MPa and 9.2%, respectively. The standard deviations of the YS, UTS and EL were 7.5 MPa, 3.5 MPa and 0.3%, respectively. The CP steels used exhibited a continuous yielding behavior, with uniform and high elongation. It could be considered that the galvanized CP steels possessed excellent engineering mechanical properties.

To shed more light on the characteristics of the LME cracks of Zn-coated CP steels prepared with different welding currents, the LME cracks have been classified into four categories according to the A/SP standard. The crack at the center of the weld is type A. A type B crack is observed outside the electrode indentation zone. Type C refers to an interfacial surface crack that occurs between the two steel plates. The LME crack at the shoulder of the weld is defined as type D.

**Figure 3:**
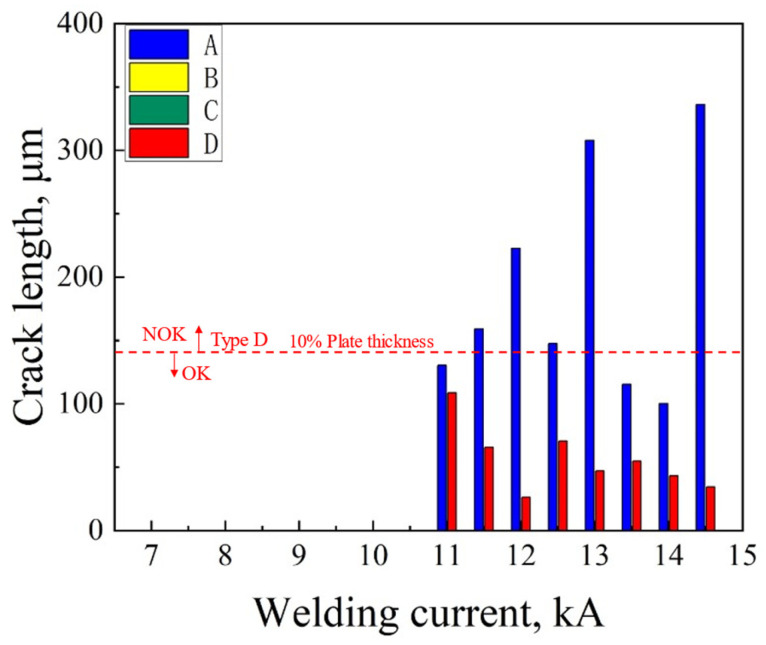
Statistical length of the LME cracks observed by the layer grinding and polishing of the sample with different welding currents.

**Figure 4:**
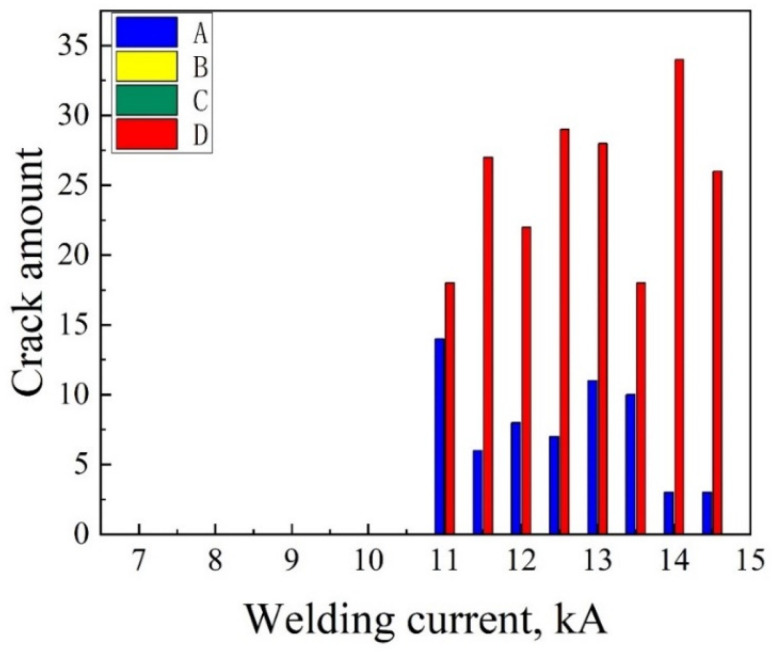
Statistical amounts of various types of LME cracks in the samples welded with different welding currents.

The lengths and amounts of different kinds of LME cracks with various welding currents were measured and counted, and the results are shown in Figure 3 and Figure 4. The ordinate of Figure 3 indicates the maximum length of each type of crack that could be observed in the entire cross-section of the samples. Similarly, the ordinate of Figure 4 is the total amount of all sorts of cracks counted in the entire cross-section of the samples. The abscissas of Figure 3 and Figure 4 represent the samples corresponding to the 16 sets of welding parameters from 7.0 kA to 14.5 kA. In Figure 3, “OK” indicates that the length of a type D crack complies with the acceptable criteria required in the A/SP standard. “NOK”, which is an abbreviation for “No OK”, denotes that the length of the type D crack did not meet the requirements of the A/SP standard. For all the welded joints, neither type B nor type C cracks were found under each welding current. In the samples below expulsion, no LME crack was observed. However, type A and D cracks could be found in the samples when the welding current was increased to 11.0 kA and expulsion occurred (defined as at expulsion). The longest type A crack appeared at the welding current of 14.5 kA, with the length of 336.1 μm. As for type D cracks, all of them were below 10% plate thickness (140 μm). The longest type D crack was 108.5 μm at the welding current of 11.0 kA. Some previous studies have revealed that tensile stress during the RSW process could exacerbate the propagation of cracks [38,47]. On the contrary, compressive stress could play a certain inhibitory role in the generation and development of LME cracks [48]. In our previous study [49], it was found that only tensile stress was generated at the center of the weld joint during the welding and holding processes, and tensile and compressive stresses coexisted at the shoulder of the weld joint during the welding process, yet compressive stress always played a role in the holding process. Therefore, the lengths of type D cracks were generally shorter than those of type A cracks. Figure 4 shows that the amount of type A cracks decreased when increasing the welding current, while the amount of type D cracks remained at a high level. In light of the A/SP standards, type A cracks in the conditions of at expulsion and above expulsion were not necessarily evaluated, and type D cracks, below 10% of the plate thickness, were acceptable. It could be seen that the LME susceptibility of Zn-coated CP steel was low, meeting the generally acceptable requirement of LME cracks in the A/SP standard at different welding currents.

**Figure 5:**
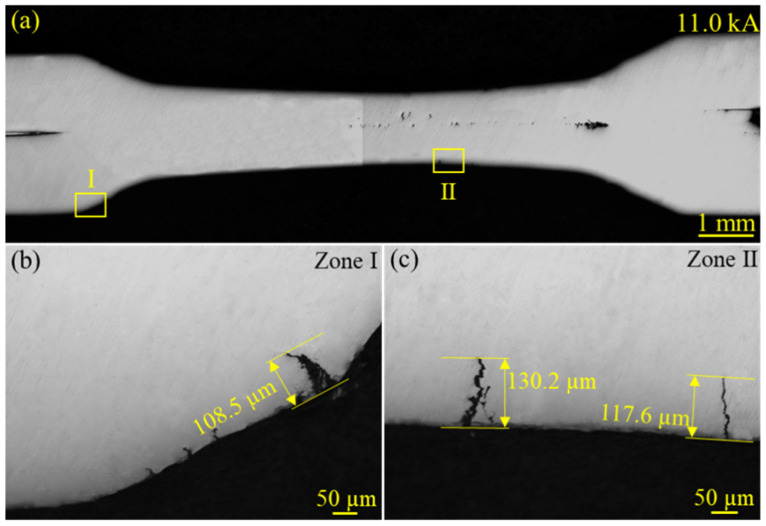
Metallographic images of the longest type D cracks in all samples: (**a**) the section image with the longest type D crack, (**b**) the morphology details of the type D crack in zone I is indicated with a yellow rectangle in (**a**); (**c**) the morphology details of the type A crack in zone II are indicated with a yellow rectangle in (**a**).

Figure 5a shows the typical observation results of LME cracks in the sample welded at 11.0 kA. Clearly, two types of LME cracks were detected. The length of the LME crack was the vertical distance between the surface and the crack tip. The morphological details of the type D crack are shown in Figure 5b. This type D crack was the longest type D crack found under all welding parameters, with a length of 108.5 μm. The longest type A crack under this welding current is shown in Figure 5c, with a length of 130.2 μm.

**Figure 6:**
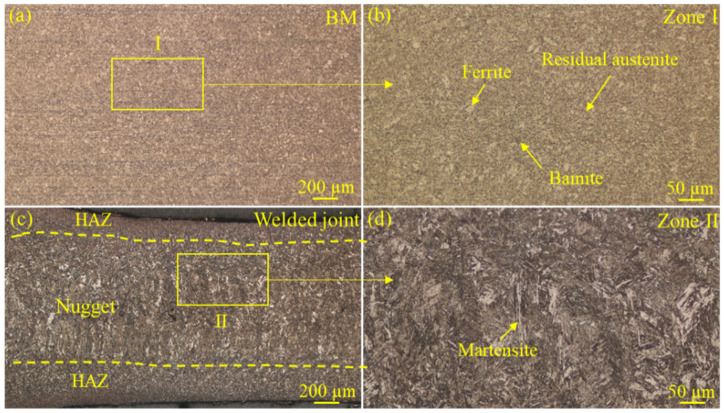
Microstructure of different characteristic zones of the welded joint at the welding current of 11.0 kA: (**a**,**b**) the microstructure of the BM, (**c**,**d**) the microstructure of the nugget and the heat-affected zone.

Figure 6 shows metallographic images of the microstructure of the base metal (BM) and the nugget of the welded joint at the welding current of 11.0 kA. As shown in Figure 6b, the microstructure of the BM consisted of ferrite, bainite, and a small amount of residual austenite. Figure 6c shows the part of the nugget and heat-affected zone where the welded joint was in contact with the copper electrode. Owing to the excellent thermal conductivity of the copper electrode and the high cooling rate, the formed microstructure could be refined. Figure 6d shows the microstructure at the nugget, which was the lath martensite formed by the rapid cooling of the original microstructure after high-temperature austenitizing. Some studies found that materials with an austenitic structure could possibly produce a higher susceptibility to liquid metal embrittlement compared to materials mainly composed of martensite and ferrite [50]. However, many studies have offered different viewpoints from that opinion in recent years. Jung et al. [51] reported that the LME phenomenon occurred in not only the austenitic steel, but also in the martensitic and ferritic steels. Pant et al. [34] studied the LME susceptibility of three kinds of martensitic steels with different compositions but the same strength. They found that the levels of LME susceptibility of the three martensitic steels were not the same, and deemed that this phenomenon was related to the grain boundary properties of the steel. These results indicate that the microstructure of steel could possibly produce a certain effect on the LME susceptibility of steel, but this effect was not decisive, implying that there are other more important influential factors.

**Figure 7:**
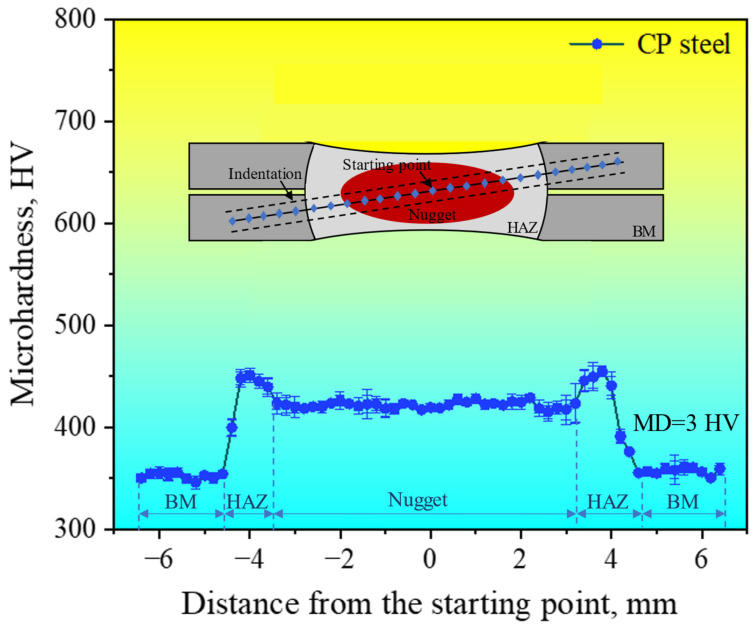
Microhardness distribution of the welded joint at the welding current of 11.0 kA. The inset reflects the schematic of the area taken for the microhardness measurement.

Currently, the widely accepted opinion is that the LME susceptibility of steel increases when increasing the steel strength. For example, AHSSs such as QP steel, TWIP steel, and TRIP steel exhibit relatively high LME susceptibility. In contrast, the LME phenomenon is rarely found during the resistance spot-welding process of traditional low-carbon steel. Hardness can reflect the strength of steel to a certain extent. Therefore, it is necessary to measure the hardness of the welded joint. The microhardness distribution of a welded joint cross-section at the welding current of 11.0 kA is shown in Figure 7. The microhardness of the BM was about 350 HV. The microhardness of the heat-affected zone (HAZ) was the highest, reaching about 450 HV. The microhardness of the nugget was slightly lower than that of the heat-affected zone, with the value about 420 HV. The mean deviation (MD) of the microhardness was about 3 HV, reflecting the stability of the microhardness of various characteristic zones. The distribution of microhardness is consistent with the microstructure at different characteristic zones, as shown in Figure 5. The microstructure of the base metal mainly comprised ferrite, bainite, and a small amount of residual austenite, revealing a low hardness, while the nugget and heat-affected zone were composed of martensite because of the higher temperature and faster cooling rate in the resistance spot-welding process, and thus the hardness was higher. In general, the microhardness distribution of the welded joint was reasonable, indicating that the microstructure in each zone of the welded joint was relatively stable and uniform.

**Figure 8:**
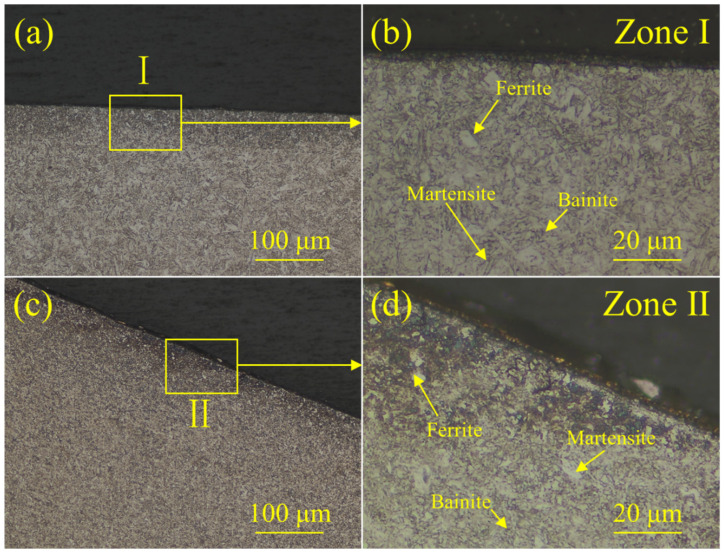
Microstructures of different characteristic zones of the welded joint at the welding current of 11.0 kA: (**a**,**b**) the microstructure of the welded joint center, (**c**,**d**) the microstructure of the welded joint shoulder.

The corroded center and shoulder regions of the welded joint were observed and are shown in Figure 8. The ferrite, bainite martensite and bainite were all found in these regions. However, it was hard to determine the precise contents of these three phases. Lima et al. [52] studied the microstructure of CP1100 steel, and found that the ferrite and bainite contents were around 56.3 ± 4.0% and 21.1 ± 4.1%, respectively. Additionally, some studies had already compared the LME susceptibilities of steels with different microstructures [36,53] and deemed that the type of microstructure was not the crucial factor influencing LME susceptibility.

**Figure 9:**
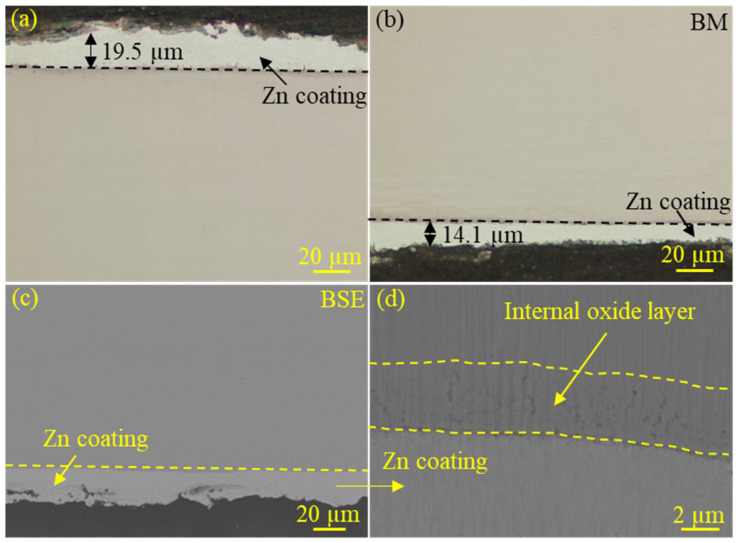
Microstructure of the galvanized CP steel and thickness of Zn coating: (**a**,**b**) the metallographical images of the CP steel with Zn coating, (**c**,**d**) the backscattered electron (BSE) images of the CP steel with Zn coating.

Figure 9a,b shows the metallographic images near the surface of the sample section. Since the Zn coating could easily be damaged during grinding and polishing, the coating thickness shown in the figure was non-uniform. In actual production, the thickness of the Zn coating could be controlled to be approximately 20 μm by adjusting the hot-dip galvanizing process. Figure 9c shows the status of the coating and the steel substrate at the base metal as revealed via a scanning electron microscope. A more detailed observation could be made through local magnification, as shown in Figure 9d. By controlling the dew point of the protective atmosphere during the annealing process, an internal oxidation layer could be formed on the subsurface of the steel plate, and oxides were distributed along the grain boundaries. The internal oxide layer can be clearly observed in Figure 9d, where an obvious element segregation occurred in the near surface of the substrate used for the CP steel matrix. According to the technological process of hot-dip galvanizing, the steel plates needed to be annealed before being immersed in a crucible filled with molten zinc to improve the platability of the steel plates. Shibli et al. [54] also found that some elements in the steel could be selectively oxidized during the annealing process before the hot dip galvanizing process.

**Figure 10:**
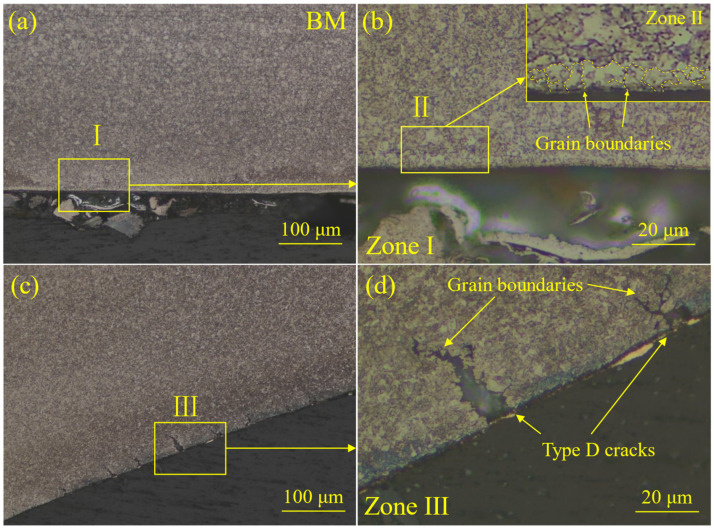
Microstructure of the galvanized CP steel: (**a**) the metallographic image of the near-side surface of BM, (**b**) a metallographic image of the enlarged zone I in (**a**), (**c**) a metallographic image of the shoulder region of the welded joint, (**d**) a metallographic image of the enlarged zone III in (**c**).

To determine the position of the internal oxide layer, the near-side surface of the BM of the galvanized CP steel and type D cracks of the welded joint were corroded and observed via OM, and the results are shown in Figure 10. The presence of grain boundaries could be clearly observed in Figure 10b. Furthermore, Wu et al. [55] presented the view that oxygen entered the steel matrix through the channels of the grain boundaries, and the Si at the grain boundaries was oxidized to SiO_2_. Additionally, a previous study found that the internal oxides distributed at the grain boundaries of dual-phase steel could affect the formation of LME cracks [56]. The type D cracks are shown in Figure 10c,d. The grain boundaries could be observed at the crack tips, which proves that the LME cracks propagated along the grain boundaries. Based on the above evidence, it could be reasonably determined that the internal oxide layer was distributed at the grain boundaries near the near surface of the BM.

**Figure 11:**
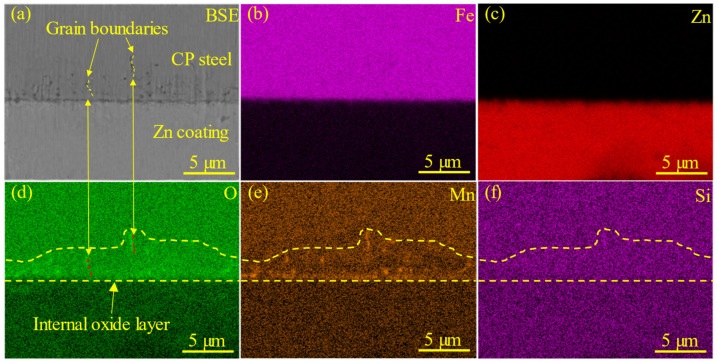
Microstructure and element distribution of the sample at the welding current of 11.0 kA: (**a**) BSE image of the microstructure adjacent to the Zn coating and internal oxidation layer of the steel substrate, (**b**–**f**) the corresponding EDS mapping results taken to highlight the element distributions of Fe, Zn, O, Mn and Si, respectively. The grain boundaries could be traced based on the backscattering mode, and are correspondingly pointed out in (**a**).

In order to shed more light on the element segregation near the surface of the steel matrix, the sample was analyzed using energy dispersive spectrum (EDS). Figure 11 present the backscattered electron (BSE) and EDS images, which illustrate the distribution of elements in the near-side surface region of the welded joint at the welding current of 11.0 kA. Obvious element segregation could be observed in the base BM of the sample. The corresponding energy spectrum results highlight the segregation of Mn and O elements, as shown in Figure 11d,e. Moreover, the Mn and O elements exhibited a tendency of simultaneous segregation, forming an internal oxide layer near the surface of the Zn-coated CP steel sheet. Some complex oxides formed by Si and Mn with O could reduce the wettability of liquid Zn to the steel matrix [57]. According to the previous findings of our research group [58,59], the internal oxide layer and the oxides distributed at the grain boundaries could indeed reduce the penetration of the liquid Zn, and tend to inhibit the formation of LME cracks. Therefore, the oxides at the grain boundaries of galvanized steel could suppress the growth of LME cracks during the resistance spot-welding process.

**Figure 12:**
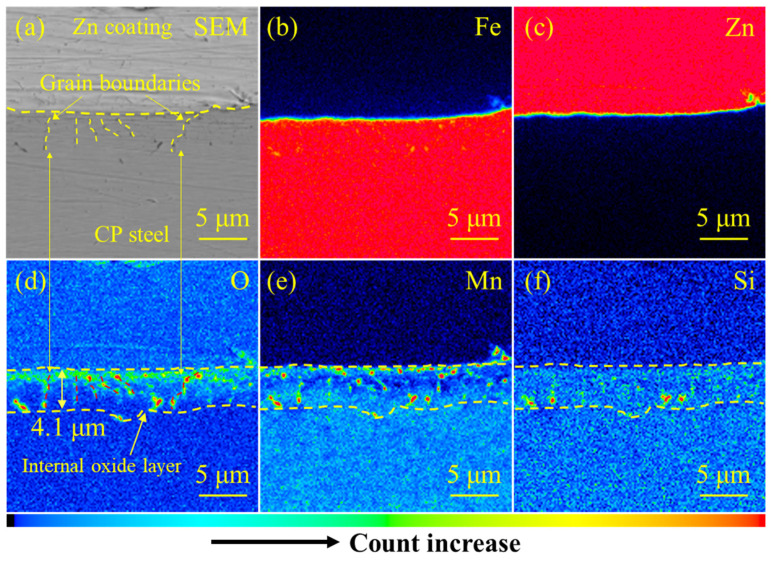
EPMA results for the collected region in the BM of galvanized CP steel: (**a**) SEM image of the Zn coating, and (**b**–**f**) element distributions of Fe, Zn, O, Mn, and Si, respectively. The grain boundaries could be traced based on the backscattering mode, and are correspondingly highlighted in (**a**).

To acquire more distinct information about element distribution around the internal oxide layer, an electron probe micro-analyzer (EPMA) was adopted. As shown in Figure 12, the results are more explicit, revealing the enrichment of the O element. Meanwhile, the simultaneous distribution trends of Si and Mn elements are shown in the internal oxide layer. The element distribution patterns of Fe, Zn, O, Mn and Si are illustrated in Figure 12b–f. The results indicate that the segregation of O, Mn, and Si occurred near the surface of CP steel, which is in accordance with the results obtained by EDS (see Figure 11). Moreover, the depth of this protective layer was measured, with the depth being about 4.1 μm. The liquid Zn could be inhibited by the internal oxide layer, thus reducing the tendency toward LME occurrence.

In recent studies, most researchers considered that LME cracks occurred at and propagated along grain boundaries. Generally, with the aim of enhancing the adhesion between the steel and the Zn coating, an annealing treatment was applied prior to the process of hot-dip galvanizing. Miyata et al. [60] proposed that Si and Mn elements in steel tended to form oxides at the grain boundaries during the annealing process. It could be more difficult for liquid Zn to penetrate into the matrix because of these internal oxides. On the other hand, Fe–Zn intermetallic compounds, which could continuously erode and embrittle the steel matrix, were hardly formed at the grain boundaries [58]. Bhattacharya et al. [61] studied the advanced high-strength steels with different Si contents, and found that higher LME susceptibility occurred in the steels with high Si contents. Therefore, the LME susceptibility of the Zn-coated CP steel could be successfully decreased when the Si and Mn elements in the grain boundaries of the CP steel matrix are simultaneously oxidized.

**Figure 13:**
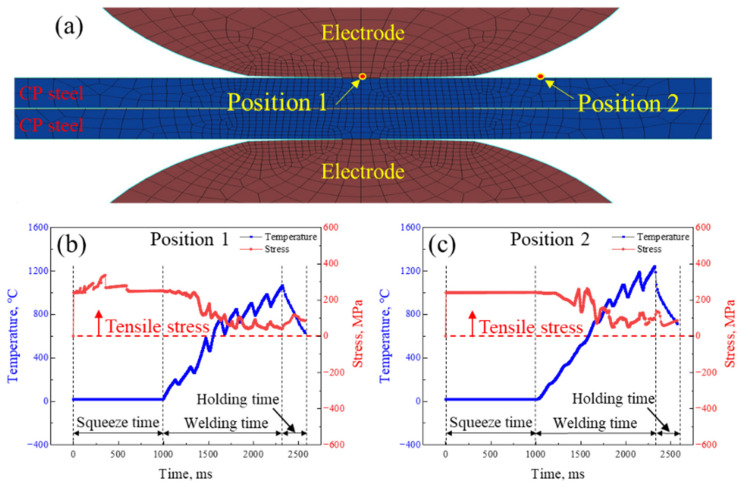
The thermal and stress simulation of the galvanized CP steel during the in RSW process: (**a**) the mesh graph depicting the region of the welding spot during the simulated RSW process, (**b**) the temperature and stress distribution at position 1 in (**a**) for the whole welding process of the welded joint, (**c**) the temperature and stress distribution at position 2 in (**a**) for the whole welding process of the welded joint.

Generally, LME cracks were formed during the RSW process, and the stress in the RSW process played a crucial role in the formation of LME cracks [47,62]. Through the SORPAS software, the temperature and stress distributions during the welding process were correspondingly simulated. The temperature and stress histories at the center and shoulder of the joint during the RSW process were obtained. Figure 13 presents the temperature–time and stress–time curves at the center of the joint (Figure 13b) and at the shoulder of the joint (Figure 13c). The fluctuation of the temperature during the welding time stage was due to the application of eight stages of gradually increased inclined pulse currents, with a duration of 130 ms and a cooling time of 40 ms. Both positions 1 and 2 were affected by tensile stress throughout the whole process. Besides this, DiGiovanni et al. [63] found that external tensile stress was a necessary condition for the formation of LME. CP steel was affected by external tensile stress, which was conducive to the formation of LME cracks throughout the entire RSW process.

## 4. Conclusions

Through the characterization of cracks formed in the welded joints under different welding currents, the LME susceptibility of the galvanized complex phase steel was evaluated. In addition, the formation of the LME cracks was discussed based on the microstructure analysis and element distribution in the grain boundaries. The conclusions are as follows:

(1) The main microstructure of CP steel comprised ferrite and bainite, with the microhardness of about 350 HV. On the contrary, the microstructure in the nugget produced via resistance spot-welding included martensite, with a microhardness of about 420 HV. The microhardness of the heat-affected zone (HAZ) was about 450 HV. The mean deviation of the cross-section microhardness for the various characteristic zones of the welded joint was about 3 HV;
(2) Expulsion occurred in the Zn-coated galvanized CP steel when the welding current increased to 11.0 kA. Simultaneously, the LME cracks began to appear from 11.0 kA. The longest LME crack appeared at the welding current of 14.5 kA, which was determined as a type A crack with a length of 336.1 μm. The longest type D crack with a length of 108.5 μm was also produced with the welding current of 11.0 kA. The longest type D crack was only 7.8% of the plate thickness, less than 10% of the plate thickness, thus meeting the requirement of the A/SP standard;
(3) There was an internal oxidation layer along the grain boundaries of the galvanized CP steel matrix, which was favorable to reducing its LME susceptibility. The composition of the CP steel and internal oxidation layer could result in low LME susceptibility in the galvanized CP steel.

## Figures and Tables

**Table 1:** Chemical composition of the CP steel (wt. %).

C	Mn	Cr	Mo	Si	P	S	Fe
>0.15	>2.0	>0.3	>0.1	>0.1	<0.03	<0.01	Bal.

## Data Availability

The original contributions presented in the study are included in the article; further inquiries can be directed to the corresponding authors.
